# Hypertensive Encephalopathy: A Case of a Male Who Bit Off His Fingers

**DOI:** 10.7759/cureus.1334

**Published:** 2017-06-11

**Authors:** Carole H Kim, Saba Syed

**Affiliations:** 1 Psychiatry, Olive View, UCLA Medical Center

**Keywords:** hypertensive encephalopathy, hypertensive crisis, altered mental status, agitation, psychosis, behavior, consultation-liaison psychiatry

## Abstract

Although altered consciousness and other neurologic manifestations are frequently seen in hypertensive encephalopathy, behavioral and psychotic symptoms are rarely seen. We describe a patient with no previous psychiatric history who was admitted for hypertensive crisis. A few days after admission, his blood pressure remained uncontrolled and he started to exhibit episodes of confusion, agitation, and psychosis. During one particular episode, he overcame multiple staff members and physical restraints to bite off two of his fingers without any signs of pain. Brain computed tomography (CT) was notable for possible posterior cerebral and cerebellar edema. His confusion and agitation gradually resolved with successful blood pressure management. This is the first reported case of extreme, agitated behaviors and auditory hallucinations in a patient with hypertensive crisis.

## Introduction

Elevated blood pressure is considered as a potential predisposing factor as well as a precipitating factor for acute changes in mental status. The pathogenesis of the link between chronic hypertension and altered mental status (AMS) is uncertain, but proposed mechanisms include: 1) associated brain atrophy leading to cognitive impairment and 2) atherosclerosis resulting in brain hypoperfusion and cellular hypoxia [1]. AMS is also a possible complication of hypertensive crisis (i.e. sudden elevation of blood pressure above 180/120) secondary to cerebral edema, which occurs when the autoregulation of cerebral blood flow is overwhelmed and cerebral vasodilation ensues. Hypertensive encephalopathy is the general term for the presence of altered consciousness and other neurologic findings in the context of a hypertensive crisis [2]. If there is clinical and neuroimaging evidence of vasogenic cerebral edema, that is completely reversible following rapid treatment of the underlying disease, the more specific diagnosis of posterior reversible encephalopathy syndrome (PRES) can be made. PRES is most commonly associated with arterial hypertension but has also been linked with renal disease, pre-eclampsia, sepsis, autoimmune disorders and immunosuppressive and cytotoxic therapy [3]. For both hypertensive encephalopathy and PRES, behavioral disturbances and psychotic symptoms have been reported sparingly.

## Case presentation

We present a case of a 40-year-old Hispanic male with no prior psychiatric history and a medical history of hypertension and end-stage renal disease (ESRD). He was recommended to visit the emergency department (ED) after he was dizzy with a systolic blood pressure in the 80s following an outpatient dialysis session. By the time he presented in the ED, his blood pressure was 209/125 mmHg with the resolution of his dizziness and he was admitted for hypertensive crisis. The patient reported intermittent non-compliance with his home clonidine. In addition to starting his home hypertensive medications in the ED, ciprofloxacin, and metronidazole were initiated because of his complaint of diffuse abdominal pain for two days and concern for enteritis on computed tomography (CT) of the abdomen.

On hospital admission day two, the patient began displaying episodes of confusion and agitation (Table 1). He pulled out his intravenous lines, climbed onto the windowsill, and wrote “help” on the window. On day four, he reported hearing voices of his boss’s wife and seeing a person from his dialysis center walking around. The next day, he was calm and pleasant during morning rounds but then bit his right thumb a few minutes later. Despite several staff members holding him down, he bit off his distal thumb and did not appear to be in any pain. He was placed in hard restraints and haloperidol 5mg was administered intramuscularly (IM). The patient became calmer and asked the staff to forgive him. However, within a few seconds, he bit off his distal left index finger. Then, he tried pulling out his permacath with his teeth and spat at staff. He required three administrations of lorazepam 2mg IM before his agitation resolved. He appeared confused throughout this incident and could not explain why he was agitated. Later that night, he verbalized that the “devil” bit his fingers. Plastic surgeons were unable to reattach the avulsed sections of his fingers. He continued to be confused and agitated on day six, but to a lesser degree compared to the previous day. On day seven, the patient stated that he was “dreaming” when he was biting his fingers and was unable to stop himself. For the remainder of the hospitalization, his mental status was improved and stable except for occasional mild agitation.

**Table 1:** Daily medical and behavioral overview

Day	Median blood pressure (mmHg)		Serum labs							Dialysis	Mental Status/Behavior Changes
	Systolic	Diastolic	Urea nitrogen (mg/dL)	Creatinine (mg/dL)	Glucose (mg/dL)	Sodium (mEq/L)	Total calcium (mg/dL)	Magnesium (mg/dL)	Phosphorus (mg/dL)		
0	208	129	35	8.10	98	142	9.0			X	
1	166	109									
2	166	106	70	11.59	80	137	8.5	2.8	3.6		single episode of confusion and agitation overnight
3	186	111								X	alert and oriented, no behavioral issues
4	161	91	36	9.57	100	133	8.0	2.4	3.6	X	visual and auditory hallucinations, episodic agitation overnight
5	184	96	20	7.04	86	136	7.9	2.2	4.2		episodic confusion, delusions, extreme agitation and biting off fingers
6	178	105	27	10.15	120	132	8.5	2.3	4.5	X	less severe confusion and agitation
7	162	88	18	8.59	79	139	8.6	2.5	4.2		alert and oriented, no behavioral issues
8	147	88								X	mild agitation
9	169	93	10	7.32	76	142	8.1	2.3	4.8		no behavioral issues
10	139	78	11	0.72	105	140	7.2	1.8	2.1		mild agitation
11	135	75								X	mild agitation
12	163	94	15	7.70	76	140	8.4	2.3	4.2		mild agitation
13	144	90	18	0.40	81	134	8.0	2.2	4.7	X	return to baseline mental status (alert, oriented, calm, pleasant, cooperative)
14	138	88									

The patient denied any headaches, dizziness, nausea, dyspnea, chest pain, weakness, or vision changes. His systolic blood pressure was most consistently in or near the critical range (i.e. above 180 mmHg) between days one-six (Table 1) and required intravenous hydralazine and labetalol daily during this time period. His other vital signs were within normal range except for occasional tachycardia and his neurological examination was unremarkable. There were no significant changes in his electrolyte or glucose levels (Table 1). However, his laboratory results showed evidence of his ESRD including elevated blood urea nitrogen and creatinine levels which fluctuated with his thrice-weekly dialysis (Table 1), chronic anemia, and secondary hyperparathyroidism. The patient had no known family psychiatric history. He denied any illicit drug use. Urine toxicology was not performed due to oliguria. The patient scored a 16/30 on Montreal Cognitive Assessment on day four. Brain non-contrast computed tomography (CT) on day seven showed possible cerebellar fullness with narrowing of the sulci and subtle asymmetry posteriorly within the parietal and occipital lobes. Electroencephalogram (EEG) on day 10 was consistent with mild or resolving encephalopathy and possible drug effects.

His blood pressure medication regimen was optimized by starting labetalol, increasing losartan, continuing nifedipine, and discontinuing metoprolol and clonidine. Ciprofloxacin and metronidazole were stopped after six to seven days as the most likely etiology of his abdominal pain was viral gastroenteritis considering the absence of any other gastrointestinal symptoms and resolution by day three. He received risperidone for four days as well as haloperidol and lorazepam as needed for control of his agitation. On the day of discharge (day 14), the patient’s blood pressure was better controlled (systolic: 134–144 mmHg, diastolic: 80–93 mmHg) and he was fully oriented, calm, and cooperative. He did not remember any of his episodes of agitation but was remorseful for his actions and planned to be fully compliant with his blood pressure medications. At his one-week follow-up visit and multiple visits for urological issues over the next four months, his blood pressure was well-controlled and he did not display any symptoms present during his altered state.

## Discussion

The initiation of the patient’s altered mental status after persistent hypertension in the critical range, resolution following successful management of hypertensive emergency and CT brain findings of possible edema suggest that his AMS was most likely secondary to hypertensive encephalopathy. PRES should also be considered due to the parieto-occipital distribution of his cerebral edema, acute onset, and reversibility of his AMS, and presence of two commonly associated risk factors (renal dysfunction and sudden elevation in blood pressure) [3]. Unfortunately, a magnetic resonance imaging (MRI) was not performed for radiologic confirmation of vasogenic edema. The patient’s intermittent non-compliance with clonidine was likely responsible for rebound hypertension and tachycardia as its abrupt cessation is proposed to increase sympathetic activity [4].

Other organic etiologies are less likely to be the primary cause of his AMS. Excited catatonia was ruled out due to the waxing and waning nature of his confusion, severity of his agitation and absence of other signs of catatonia (e.g. mutism, rigidity, stereotypy). Although the patient had a brief episode of hypotension prior to admission, it is unlikely that acute cerebral hypoperfusion was the predominant etiology considering his AMS began on day two and gradually progressed. The clinical course of his suspected viral gastroenteritis did not coincide with his acute change in mental status; his abdominal pain started two days before admission and resolved by day three. He was afebrile throughout his hospitalization and there was no evidence of any systemic infections. Common metabolic etiologies of AMS were eliminated by close monitoring of his electrolyte and glucose levels, which did not correlate with any of his mental status changes. While a frequent cause of AMS is medication-induced, the only new medications that were started during his hospitalization were ciprofloxacin, metronidazole, hydralazine, and labetalol. Other signs of neurotoxicity would likely have been seen in ciprofloxacin or metronidazole-induced encephalopathy. Also, hydralazine and labetalol have not been associated with any psychiatric symptoms in the recent literature. Lastly, the absence of any seizure-like movements or any epileptic discharges on (electroencephalogram) EEG makes post-ictal confusion or psychosis unlikely.

Out of all the possible organic causes of his AMS, his history of ESRD was explored with the most scrutiny. ESRD is known to have many neuropsychiatric consequences including changes in mood, psychomotor activity, behavior, and cognitive function via multiple mechanisms. These mechanisms include poor uremic toxin clearance, high parathyroid hormone levels, sodium and calcium imbalance and decreased catecholamine synthesis [5]. While it is possible that his renal failure was a contributing factor to his presentation, there was no evidence of any significant acute changes in his kidney function prior to or during hospitalization. He was compliant with his outpatient dialysis treatments and received dialysis three times per week as an inpatient. The fluctuations in his blood urea nitrogen and creatinine seemed to be associated with the timing of his inpatient dialysis sessions and did not correlate with the occurrence or severity of his confusion or agitation. Although there was an elevation in his blood urea nitrogen and creatinine on the day of his initial episode of confusion and agitation, his blood urea nitrogen and creatinine were relatively low on day five, which was the day he bit off his fingers and spat at staff members. The absence of recurrence of AMS during subsequent medical follow-ups also makes it improbable that ESRD was the primary etiology of his acute changes in mental status.

The most common clinical manifestations of hypertensive encephalopathy and PRES are disorders of consciousness (from confusion to coma), seizure, visual disturbances, and headache [2-3]. Visual disturbances occur because of occipital lobe involvement and can present in many forms, including visual hallucinations as with our patient [3,6]. Our patient’s presentation was unusual for a few reasons. In addition to visual hallucinations, he displayed auditory hallucinations and delusions. Although present for only a brief period, these symptoms suggest that the temporal and frontal lobes may have also been affected [7]. While there are no reports of auditory hallucinations, there have been single case reports of tactile hallucinations and delusional infestation in PRES patients [8-9]. Another unique aspect of his presentation was the severity of his aggression during the finger biting episode. This episode was notable for his high pain tolerance, ability to overcome physical restraints and unresponsiveness to verbal commands. While agitation has been reported in patients with hypertensive encephalopathy in a few case reports, these reports only mentioned agitation briefly and did not elaborate on the severity of the agitation [6,10]. This case highlights the wide spectrum of neuropsychiatric symptoms that can occur as a consequence of sudden, sustained elevations in blood pressure. And, the absence of residual symptoms suggests that these symptoms cannot be explained by underlying psychiatric pathology. 

## Conclusions

To our knowledge, this is the first described case of extreme, agitated behaviors and auditory hallucinations in the context of hypertensive encephalopathy and/or PRES. While this patient may have been predisposed to altered mental status (AMS) due to his chronic hypertension and renal disease, it is unclear why his presentation was primarily psychiatric in nature. Despite the unique presentation, we were able to successfully manage his symptoms by aggressively treating the underlying medical etiology and symptomatically managing with antipsychotic treatment to prevent significant harm.
